# Associations of Different Adipose Tissue Depots with Insulin Resistance: A Systematic Review and Meta-analysis of Observational Studies

**DOI:** 10.1038/srep18495

**Published:** 2015-12-21

**Authors:** Mingzhi Zhang, Tian Hu, Shaoyan Zhang, Li Zhou

**Affiliations:** 1 Department of Epidemiology, School of Public Health, Medical College of Soochow University, Suzhou, Jiangsu, China; 2 Jiangsu Key Laboratory of Preventive and Translational Medicine for Geriatric Diseases, School of Public Health, Medical College of Soochow University, Suzhou, PR China; 3 Department of Epidemiology, School of Public Health and Tropic Medicine, Tulane University, New Orleans, Louisiana, USA; 4 Department of Neurology, The First Affiliated Hospital of Soochow University, Suzhou, Jiangsu, China

## Abstract

Fat distribution is strongly associated with insulin resistance, a risk factor for type 2 diabetes and cardiovascular diseases. However, associations of different adipose tissue depots or/and obesity indices with insulin resistance have not been systematically evaluated. In this study we examined associations of different adipose tissue depots/obesity indices with insulin resistance, as measured by homeostatic model assessment of insulin resistance (HOMA-IR) in observational studies. A total of 40 studies with 56 populations and 29 adipose tissue depots/obesity indices were included in the meta-analysis. There were strong correlation between HOMA-IR and visceral fat mass (r = 0.570, 95% confidence interval(CI): 0.424~0.687), total fat mass (r = 0.492, 95%CI: 0.407~0.570), body mass index (r = 0.482, 95%CI: 0.445~0.518) and waist circumference (r = 0.466, 95%CI: 0.432~0.500), except lower extremity fat (r = 0.088, 95%CI: −0.116~0.285). Sample size, diabetic status, gender, mean of body mass index, and race contributed to heterogeneity of these associations. This study showed a positive correlation between insulin resistance and most adipose tissue depots/obesity indices, and the strongest association is for visceral fat mass.

Insulin resistance, a key determinant of metabolic syndrome^1^,^2^,^3^, is an important risk factor for type 2 diabetes^1^ and cardiovascular diseases^4^,^5^. Adiposity, a major determinant of insulin resistance^6^,^7^,^8^, and its distribution measures have been shown to be associated with insulin resistance by a number of studies^7^,^9^,^10^,^11^,^12^,^13^,^14^,^15^,^16^,^17^,^18^,^19^. However, to what extent various adipose tissue depots and obesity indices are associated with insulin resistance has not been systematically evaluated.

Among insulin resistance indices, homeostatic model assessment of insulin resistance (HOMA-IR) is the most commonly used in population studies^20^,^21^. In this meta-analysis, we systematically examined the associations of HOMA-IR with different indices of adiposity and body fat distribution, such as body mass index (BMI)^12^,^13^,^14^, waist circumference^13^,^15^,^16^, trunk fat mass^17^,^18^,^19^, visceral fat^22^,^23^, and total fat mass^23^,^24^ to identify which of the adipose tissue depots/obesity indices has the best association with insulin resistance.

## Result

### Basic characteristics of the included studies

A total of 29 adipose indices were reportedly associated with HOMA-IR. 17/29 indices were not included in the meta-analysis because they were reported only once or twice (Table 1). The remaining 12 adipose tissue depots/obesity indices that were reported more than three times were analyzed with meta-analysis.

### The correlations between HOMA-IR and the 17 adipose indices that were excluded from the meta-analysis

Apart from retroperitoneal adipose tissue and suprailiac skinfold thickness, 15/17 adipose tissue depots/obesity indices showed significant correlations with HOMA-IR (Table 1). There were significant correlations between HOMA-IR and abdominal fat, intra-abdominal fat, subscapular skinfold thickness, intraperitoneal fat ratio, and subcutaneous fat ratio.

### The correlations between HOMA-IR and the 12 adipose indices revealed by meta-analysis

11/12 adipose tissue depots/obesity indices except leg or lower extremity fat mass showed significant correlation with HOMA-IR (Table 2). The strongest correlation was for visceral fat(r = 0.570, 95%CI: 0.424~0.687), followed by total fat mass (r = 0.492, 95%CI: 0.407~0.570) and body mass index (r = 0.482, 95% CI: 0.445~0.518).

### Sensitivity analyses

No study appears to drive the pooled estimation as dropping any of the studies did not materially change the pooled estimation.

### Meta-regression analysis on correlation coefficients’ related factors

The Meta-regression analysis identified a number of factors that were associated with the correlation between adipose tissue depots/obesity indices and HOMA-IR, including sample size of population, gender, race, diabetic status and mean of BMI (Table 3). In detail, sample size of population was found to be associated with correlation between visceral fat and HOMA-IR while gender was associated with correlation between subcutaneous fat or waist to hip circumference ratio and HOMA-IR. In addition, race was associated with correlation between body mass index and HOMA-IR and correlation between waist circumference and HOMA-IR while diabetic status, mean of BMI and race is associated with correlation between hip circumference and HOMA-IR.

### Statistical tests of publication bias

No publication bias was found for the 12 indices included in the meta-analyses by Begg’s test (*P* > 0.05, Table 4). Using Egger’s test, we found that 2/12 *P* values for leg (or lower extremity fat) and trunk fat respectively, fell lower than 0.05 (Table 4).

## Discussion

This meta-analysis study is the first to assess correlation between different adipose tissue depots/obesity indices and insulin resistance. We found significant correlations between most adipose tissue depots/obesity indices and insulin resistance. Among these indices, visceral fat mass showed the strongest correlation with HOMA-IR, followed by total fat mass, BMI and waist circumference. Notably, the leg fat (or lower extremity fat) had no significant correlation with HOMA-IR. In addition, diabetic status, gender, mean BMI, and race were associated with correlation estimates in meta-regression analysis. These findings may have important clinical and public health implications for prevention and treatment of diabetes.

In this study visceral fat mass showed the strongest correlation with HOMA-IR, followed by total fat mass, BMI and waist circumference. Other studies, which were not included in this meta-analysis, also reported significant correlation between HOMA-IR and intraperitoneal fat ratio^25^, intra-abdominal fat^23^, abdominal fat^26^ and sagittal abdominal diameter^14^,^27^ with correlation coefficients around 0.5. Visceral adipose tissue appeared to be the best predictor of insulin resistance^28^,^29^,^30^, measured by the clamp technique. Kelley *et al.*^30^ reported that insulin-stimulated glucose utilization was significantly correlated with both visceral adipose tissue and deep subcutaneous adipose tissue (r = −0.61 and −0.64, respectively; both *P* < 0.001). Nevertheless, visceral fat mass and total fat mass are measured with DEXA or magnetic resonance imaging, whereas BMI and waist circumference measurements are quick and easy using simple measuring instruments. Therefore, BMI and waist circumference are probably better predictors to be used for insulin resistance for economic reasons.

In this study, factors such as diabetic status, gender, obesity status and race were found to be associated with pooled correlation estimates. Gender difference has been widely reported regarding obesity, especially central obesity. Machann *et al.*^31^ reported that females were characterized by lower visceral adipose tissue and higher subcutaneous adipose tissue. Bouchard *et al.*^32^ also described a more pronounced increase in visceral adipose tissue in men compared to women, in normal weight, overweight, and obese individuals. Differences in HOMA-IR levels in men and women (2.06 vs. 1.93, respectively; P = 0.047) may also be a contributing factor. Insulin resistance deteriorates with age in women 50 years or older, but not so in men^33^. Many women are going through menopause at 50; therefore, menopause may also contribute to insulin resistance and obesity in women of 50 years or older.

There are some limits in our study. First, we only used HOMA-IR as an index to measure insulin resistance without testing any other method; nevertheless, indexes other than HOMA-IR are not widely used. Secondly, race/ethnicity was not well defined in some of the studies included in this work. Lastly, there is always considerable heterogeneity presented in the meta-analyses. This work is no exception and we identified a few contributing factors.

In conclusion, we found significant positive correlation between most adipose tissue depots/obesity indices and insulin resistance, as measured by HOMA-IR. Visceral fat showed the strongest correlation whereas lower extremity fat had no correlation with insulin resistance. Diabetic status, gender, race/ethnicity, and mean BMI contributed to the heterogeneity of the overall estimates.

## Methods

### Literature collection

We systematically searched PubMed, Web of Science, and Dissertation Theses to identify all relevant reports that met our inclusion criteria (see below) until September 2014. “Body mass index”, “ waist circumference”, “waist to hip ratio”, “waist to height ratio”, “abdominal height”, “fat mass”, “skinfold”, “adiposity”, “adipose tissue”, “fatness”, “body fat distribution” and “insulin resistance” in Title or Abstract, as well as MeSH terms “Body Fat Distribution”, “Body Mass Index”, “ Waist Circumference”, “Adipose Tissue”, “Skinfold Thickness” and “Insulin Resistance” were used as search terms. We also performed a manual search of references cited in published original and review articles.

The inclusion criteria were as follows: (1) the study was observational, either cross-sectional or of a case-control design; (2) conducted in humans; and (3) correlation coefficients between HOMA-IR and fat indices and their variance were reported. Studies were excluded if (1) the sample was under 19-year old; (2) the sample had chronic conditions such as cancer, heart failure, chronic kidney disease, and infectious disease. Studies of type 2 diabetes with no severe complication were included in this work.

### Data retrieval

**Figure 1:**
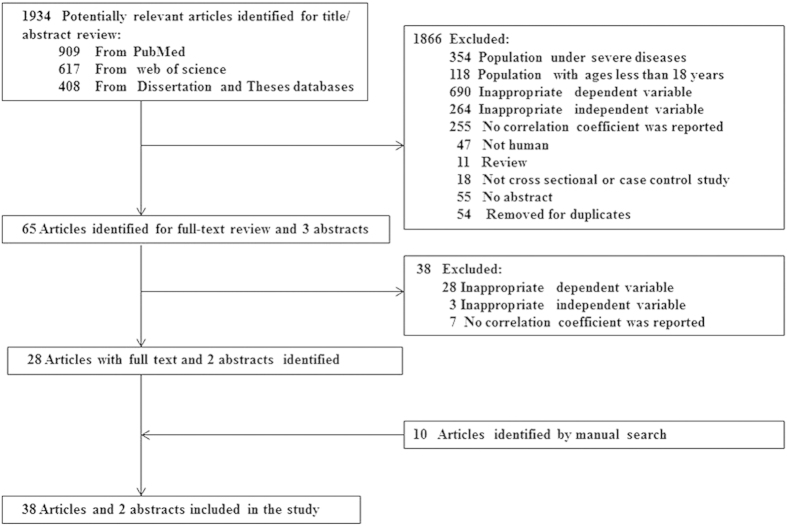
The flow chart of literature search.

All data were independently retrieved by two investigators (Zhang, M and Zhou, L) according to a standardized protocol and data-collection form. Disagreements were resolved by discussion with the third investigator (Zhang, S). First author’s name and year of publication, study design (case-control or cross-sectional), characteristics of the study subjects including sample size, mean age, mean BMI, sex, race, diabetic status, indices of adiposity, HOMA-IR transformation, and measures of associations (correlation coefficient and *P* value) were recorded. The schematic view for data retrieval is presented in Fig. 1. A total of 40 studies including 29 adipose depots or adipose indices were identified. Twelve adipose indices with at least 3 individual results were analyzed with meta-analysis.

### Data analysis

Z value from Fisher’s z-transformation of correlation coefficient by equation (1) 
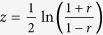
was served as effect size, and standard error of z value was calculated with equation (2) 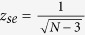. The pooled z value and 95% confidence interval was transformed into correlation coefficient and 95% CI with equation (3) 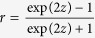. Fixed and random effect models were used to combine z values for those with more than 3 populations. Heterogeneity of z values was assessed by *I*^2^. Meta regression was performed to investigate the association between z values and sample characteristics while Begg’s and Egger’s tests were used to assess publication bias.

## Additional Information

**How to cite this article**: Zhang, M. *et al.* Associations of Different Adipose Tissue Depots with Insulin Resistance: A Systematic Review and Meta-analysis of Observational Studies. *Sci. Rep.* **5**, 18495; doi: 10.1038/srep18495 (2015).

## Figures and Tables

**Table 1:** Correlation coefficients between HOMA-IR and the 17-adipose indices that were not included in the Meta-analysis.

	Population 1			Population 2		
	n	r	P value	n	r	P value
Abdominal fat	87	0.585	<0.001	77	0.477	<0.001
Intra-abdominal fat	51	0.530	<0.05	272	0.480	<0.05
Retroperitoneal adipose tissue	51	0.110	>0.05			
Subcutaneous anterior fat	51	0.360	<0.01			
Subcutaneous posterior fat	51	0.390	<0.01			
Upper extremity fat	1579	0.460	<0.0001			
Intraperitoneal fat ratio	30	0.620	0.003			
Subcutaneous fat ratio	30	−0.550	0.011			
Liver attenuation	5291	−0.310	<0.0001			
Pericardial adipose tissue	5291	0.440	<0.0001			
Sum of the skinfold thickness	55	0.515	<0.001	55	0.254	<0.001
Subscapular skinfold thickness	55	0.595	<0.001	55	0.413	<0.01
Suprailiac skinfold thickness	55	0.288	<0.001	55	0.195	>0.05
Sagittal abdominal diameter	157	0.480	<0.0001	138	0.482	<0.001
Truncal subcutaneous fat	55	0.347	0.01			
Peripheral subcutaneous fat	55	0.296	0.028			
Thigh fat area	783	0.480	<0.0001			

**Table 2:** Pooled correlation coefficients between HOMA-IR and adipose indices and 95% confidence interval estimated with random model by Meta-analysis.

Variables	_Number of studies_	z value^a^	r value^b^
Visceral fat			
Mass	3	0.648 (0.453, 0.843)	0.570 (0.424, 0.687)
Area	9	0.438 (0.390, 0.487)	0.412 (0.371, 0.452)
Subcutaneous fat			
Mass	3	0.344 (0.149, 0.539)	0.331 (0.148, 0.492)
Area	8	0.412 (0.265, 0.558)	0.390 (0.259, 0.506)
Total fat			
Mass	7	0.539 (0.432, 0.647)	0.492 (0.407, 0.570)
Area	2	0.338 (0.188, 0.489)	0.326 (0.186, 0.453)
Fat mass percentage	6	0.436 (0.343, 0.529)	0.410 (0.330, 0.485)
Leg/lower extremity fat mass	7	0.088 (−0.117, 0.293)	0.088 (−0.116, 0.285)
Body mass index	30	0.526 (0.479, 0.574)	0.482 (0.445, 0.518)
Waist circumference	40	0.505 (0.462, 0.549)	0.466 (0.432, 0.500)
Hip circumference	10	0.436 (0.391, 0.481)	0.410 (0.372, 0.447)
Waist/Hip circumference	14	0.351 (0.290, 0.413)	0.337 (0.282, 0.391)
Waist circumference to height ratio	6	0.460 (0.402, 0.519)	0.430 (0.382, 0.477)
Leg to trunk ratio	4	−0.376 (−0.672, −0.081)	−0.359 (−0.586, −0.081)
Trunk fat mass	7	0.371 (0.186, 0.555)	0.355 (0.183, 0.504)

^a^Fisher transformation from correlation coefficient, 
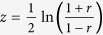
.

^b^correlation coefficient from z value, 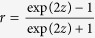.

**Table 3:** Summary of Meta-regression analysis of z value^a^.

	Visceral fat		Subcutaneous fat		Body mass index		Waist circumference		Hip circumference		Waist/Hip	
	*b*	*P* value	*b*	*P* value	*b*	*P* value	*b*	*P* value	*b*	*P* value	*b*	*P* value
Sample size												
<100	reference		reference		reference		reference		reference		reference	
100 ~ 1000	−0.136	**0.035**	−0.052	0.669	−0.131	0.148	−0.134	0.102	−0.378	0.165	0.400	0.149
>1000	0.023	−	0.0381	0.836	−0.099	0.282	−0.077	0.395	−0.368	0.173	0.351	0.204
Diabetic status												
No diabetes	reference		reference		reference		reference		reference		reference	
With diabetes	−0.013	0.928	−0.122	0.554	−0.175	0.089	−0.153	0.127	−0.097	0.097	−0.031	0.742
Mixed	0.029	0.733	−0.176	0.147	−0.070	0.284	−0.118	0.077	−0.092	**0.027**	0.016	0.833
Gender												
Male	reference		reference		reference		reference		reference		reference	
Female	0.063	0.581	0.058	0.660	0.095	0.259	0.010	0.906	−0.005	0.926	−0.179	**0.020**
mixed	−0.008	0.926	−0.402	**0.003**	0.014	0.845	−0.028	0.720	−0.040	0.496	−0.101	0.142
Method of correlation												
Pearson	reference		reference		reference		reference		reference		reference	
Pearson with Log^b^	−0.116	0.149	−0.040	0.766	−0.013	0.869	0.034	0.645	−0.025	0.719	0.054	0.519
Spearman	−0.143	0.398	0.070	0.686	0.541	0.773	0.062	0.476	−0.070	0.120	−0.008	0.917
Mean age												
<60 years	reference		reference		reference		reference		reference		reference	
≥60 years	0.004	0.968	0.219	0.292	0.021	0.847	−0.065	0.393	−0.045	0.445	−0.041	0.623
Mean BMI												
<28 kg/m^2^	reference		reference		reference		reference		reference		reference	
≥28 kg/m^2^	0.167	0.802	−0.006	0.979	0.043	0.525	0.083	0.232	−0.084	**0.027**	−0.023	0.728
Race												
Caucasian	reference		reference		reference		reference		reference		reference	
Asian	−0.021	0.770	−0.110	0.292	−0.051	0.372	−0.041	0.418	0.099	**0.016**	0.021	0.760
Other	−	−	−	−	0.604	**0.002**	0.983	**<0.001**	0.046	0.449	0.139	0.154

^a^Fisher transformation of correlation coefficient, 
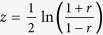
.

^b^Pearson correlation with logarithm transformation, – Sample size in the subgroup is not enough for regression analysis.

**Table 4:** Statistical tests of publication bias.

	Begg’s test		Egger’s test	
	z value	P value	bias	P value
Visceral fat	1.83	0.067	−0.5812	0.392
Subcutaneous fat	−0.96	0.337	−0.9006	0.362
Total fat	0.42	0.677	−1.1902	0.398
Fat mass percentage	0.19	0.851	−0.6610	0.617
Leg/lower extremity fat	−1.05	0.293	−6.1400	0.015
Body mass index	−0.39	0.695	−0.9814	0.296
Waist circumference	−0.08	0.935	−1.0613	0.186
Hip circumference	−0.27	0.788	−0.3973	0.763
Waist/Hip circumference	0.05	0.956	−0.0496	0.972
Waist circumference to height ratio	−0.19	0.851	−0.1624	0.911
Leg to trunk ratio	−0.68	0.497	−0.4371	0.956
Trunk fat	0.45	0.652	−3.6389	0.026
